# C5a Induces Inflammatory Signaling and Apoptosis in PC12 Cells through C5aR-Dependent Signaling: A Potential Mechanism for Adrenal Damage in Sepsis

**DOI:** 10.3390/ijms251910673

**Published:** 2024-10-03

**Authors:** Lucas Mrozewski, Sujeenthar Tharmalingam, Paul Michael, Aseem Kumar, T. C. Tai

**Affiliations:** 1 School of Natural Sciences, Laurentian University, Sudbury, ON P3E 2C6, Canada; lmrozewski@laurentian.ca (L.M.); sutharmalingam@nosm.ca (S.T.); pmichael@laurentian.ca (P.M.); akumar@laurentian.ca (A.K.); 2 Medical Science Division, NOSM University, Sudbury, ON P3E 2C6, Canada

**Keywords:** complement C5a, catecholamines, sepsis, adrenal gland, adrenal insufficiency

## Abstract

The complement system is critically involved in the pathogenesis of sepsis. In particular, complement anaphylatoxin C5a is generated in excess during sepsis, leading to cellular dysfunction. Recent studies have shown that excessive C5a impairs adrenomedullary catecholamine production release and induces apoptosis in adrenomedullary cells. Currently, the mechanisms by which C5a impacts adrenal cell function are poorly understood. The PC12 cell model was used to examine the cellular effects following treatment with recombinant rat C5a. The levels of caspase activation and cell death, protein kinase signaling pathway activation, and changes in inflammatory protein expression were examined following treatment with C5a. There was an increase in apoptosis of PC12 cells following treatment with high-dose C5a. Ten inflammatory proteins, primarily involved in apoptosis, cell survival, and cell proliferation, were upregulated following treatment with high-dose C5a. Five inflammatory proteins, involved primarily in chemotaxis and anti-inflammatory functions, were downregulated. The ERK/MAPK, p38/MAPK, JNK/MAPK, and AKT protein kinase signaling pathways were upregulated in a C5aR-dependent manner. These results demonstrate an apoptotic effect and cellular signaling effect of high-dose C5a. Taken together, the overall data suggest that high levels of C5a may play a role in C5aR-dependent apoptosis of adrenal medullary cells in sepsis.

## 1. Introduction

### 1.1. Sepsis

Sepsis, as defined by the Third International Consensus Definitions for Sepsis and Septic Shock, is a life-threatening condition caused by a dysregulated host response to infection [1]. Sepsis is characterized by high levels of inflammation and, upon disease progression, organ failure and potentially death if treatment is unsuccessful [2].

Sepsis is typically incurred through bacterial infection, with Gram-positive bacteria such as Staphylococcus aureus being the most common cause; however, fungal infections are an increasingly common cause [3]. The response to infection includes the activation, transcription, and expression of a large set of cytokines and other signaling molecules involved in promoting inflammation, activating immune tissue, and inducing immune cell chemotaxis [4,5,6]. Anti-inflammatory cytokines are also released to help modulate the effect caused by an influx of pro-inflammatory cytokines. While this response is typical of many infections, in septic environments this response extends and propagates outside the local microenvironment, inducing an uncontrolled systemic response [7]. One system associated with the promotion of the inflammatory response that is upregulated in sepsis is known as the complement system.

### 1.2. The Complement System

The complement system is composed of over 30 plasma proteins, found predominantly in an inactive, zymogen form, which are present throughout the circulatory system. The complement system is highly interconnected with three separate initiation pathways: classical, lectin, and alternative [8,9]. Each pathway is activated by its own respective stimulus, and they play an integral role in the immune system’s response to infection [8,9]. The complement system also plays a role in the mediation of inflammation, and its activation induces the release of various inflammatory cytokines, which results in both localized and systemic inflammation, depending upon the degree of complement activation [10,11]. The complement system also has important auto-immune functions such as apoptotic cell clearance, and dysfunction of this system has been proposed as a mechanism of systemic lupus erythematosus [12]. The functions of the complement system are driven primarily by its effector molecules, namely the anaphylatoxins, C3a and C5a, with C5a being the more potent of the two [13].

### 1.3. Complement C5a

C5a is a glycosylated protein that is part of the terminal complement pathway. C5a was first reported to only be a leukocyte attractant; however, it has since been shown to be involved in a broad range of immune and inflammatory functions. The two known receptors that bind C5a are complement component 5a receptor 1 (C5aR), also known as CD88, and complement component 5a receptor 2 (C5L2), also known as GPR77 [14]. C5aR is a G protein-coupled receptor (GPCR), and the interaction of C5a and C5aR results in the activation of the mitogen-activated protein kinase (MAPK)/extracellular signal-regulated kinase (ERK) pathway and induces the oxidative burst in immune cells [15,16]. In contract, C5L2 is not a GPCR, and understanding of its function remains unclear [17,18,19].

C5a levels are routinely elevated in septic individuals. In healthy human subjects, plasma levels of C5a are observed at approximately 5 ng/mL (≈0.5 nM), while in septic shock, C5a concentrations can increase to over 100 nM and remain elevated for many days following the event [17,20,21,22]. The release of C5a, while helping to generate an immune response, can also result in undesirable, damaging effects for various cell types, including both immune and non-immune cells [23,24,25,26].

C5a may also play a role in adrenal dysfunction and has been shown to impair the production of norepinephrine (NE) and dopamine (DA) in a time- and dose-dependent manner [23]. Following incubation with relatively high concentrations of C5a, there was an increase in PI and Annexin V staining, which was inhibited by the pan-caspase inhibitor Z-VAD-FMK [23]. The effects of C5a on cellular function and viability may be related to the decrease in mitochondrial function. Incubation of PC12 cells with C5a decreased mitochondrial respiration, dehydrogenase, and cytochrome c oxidase activities [27]. In addition, the mitochondrial repair protein chaperonin 60 was upregulated, demonstrating the occurrence of mitochondrial damage. In the context of secondary adrenal dysfunction, both forms of C5a receptors are expressed by the pituitary gland and the adrenal gland, and activation of these receptors is known to stimulate protein kinase signaling pathways, suggesting a possible role for C5a in the regulation of HPA [28,29,30]. Currently, the literature examining the impact of C5a on adrenal cells suggests cellular dysfunction; however, the molecular mechanisms underlying this phenotype have not yet been elucidated.

### 1.4. Primary Adrenal Dysfunction

The adrenal medulla is responsible for the synthesis of catecholamines, namely dopamine (DA), norepinephrine (NE), and epinephrine (EPI) [31]. Catecholamines are integral to the body’s response to external threats, known as the “fight-or-flight” response, which results in an increase in blood pressure and cardiac output, smooth muscle relaxation, and increased blood glucose concentrations [32,33].

Sepsis has also been shown to be associated with abnormal circulating levels of catecholamines [34,35,36]. Adrenal insufficiency is commonly observed in sepsis and may be associated with a higher mortality rate in patients [37]. Proper adrenal function has also been shown to be an important factor in the survival of mice in an endotoxin-induced sepsis model [38]. In particular, primary adrenal dysfunction, which occurs when the adrenal glands are damaged and produce inadequate levels of catecholamines, is one of the main causes of adrenal dysfunction in sepsis [39].

In vivo studies of sepsis have shown evidence of damage and dysfunction of the adrenal glands during experimentally induced sepsis. Adrenal necrosis, hemorrhage, and thrombi were identified in 37 of 46 calves suffering from bacterially caused endotoxic shock [40]. A recent study reported significant damage and apoptosis in various regions of the adrenal medulla and high levels of mRNA of inflammatory cytokines (IL-1β, IL-6, and TNF-α) in sepsis models using intraperitoneal injection of *Pseudomonas aeruginosa*, a Gram-negative bacterium [41]. Clinical evidence also shows a strong relationship between sepsis and adrenal damage. Septic patients generally have enlarged adrenal glands, approximately double the volume of the control group [42]. In addition, in a cohort of intensive care patients with septic shock, lack of adrenal gland enlargement was associated with an increase in day 28 mortality [43].

PC12 cells, derived from a rat pheochromocytoma (tumor of adrenal medullary chromaffin cells), provide an effective in vitro model of adrenal functionality and can also provide a model for analysis of neuronal function and differentiation, which is induced by exposing cells to neuronal growth factor (NGF) [44]. Numerous studies have shown that the incubation of PC12 cells with LPS induces significant cell death in both differentiated and undifferentiated PC12 cells [45,46]. This apoptotic effect was seen to be inhibited by inactivation of the caspase 3/nuclear factor kappa beta (NF-κβ) pathway via TLR-4 following pre-incubation in low concentrations of LPS [45].

### 1.5. Research Objectives and Hypothesis

As discussed, the relationship between sepsis and C5a has been well established, as has the relationship between sepsis and adrenal dysregulation. However, the molecular mechanisms by which C5a regulates adrenal cell function are poorly understood. Moreover, the cellular signaling pathways seen in C5a-treated adrenal cells have not been elucidated. In the current study, an in vitro model with PC12 cells, derived from a rat pheochromocytoma, was utilized to examine the effect of complement C5a on cellular signaling, inflammatory response, and apoptosis in rat adrenal medullary cells. It is hypothesized that exposure to septic levels of C5a will induce cell death in PC12 cells. The overall scientific objective of this study is to identify C5a cytotoxicity on PC12 cells. The secondary objective is to identify cellular signaling pathways related to the effect of C5a on PC12 cells.

## 2. Results

### 2.1. Complement C5a Induces Caspase Activation and Apoptosis in PC12 Cells

**Figure 1:**
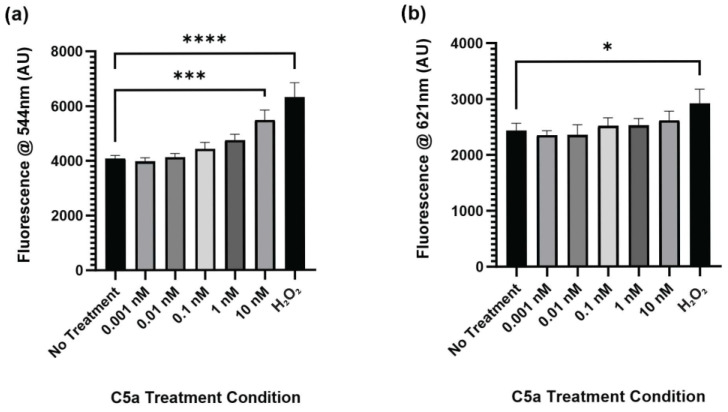
Caspase Activity and PI Staining following 30 min Treatment with C5a. (**a**) The level of caspase activation was measured by detecting the fluorescence at an excitation wavelength of 495 ± 20 nm and an emission wavelength of 544 ± 25 nm. The level of caspase activation was increased in the 10 nM C5a treatment and H_2_O_2_ positive control. (**b**) The level of PI staining was determined by measuring the fluorescence using an excitation wavelength of 586 ± 15 nm and a read wavelength of 621 ± 25 nm. The level of cell death was only elevated in the H_2_O_2_ positive control. One-way ANOVA with Dunnett’s post hoc test were used for statistical analysis. * *p* < 0.05, *** *p* < 0.001, **** *p* < 0.0001. *n* = 3.

**Figure A1:**
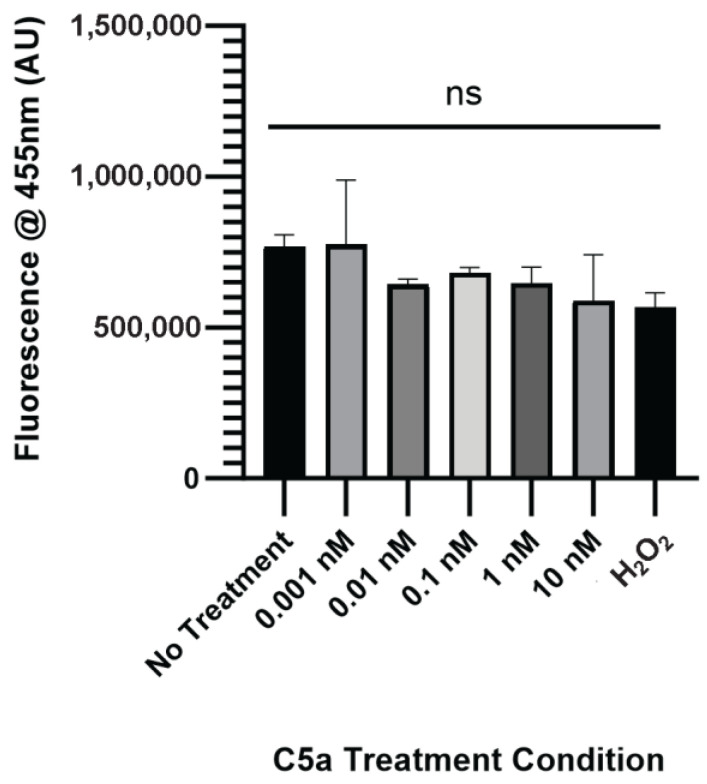
Nuclear Staining following 24 h Treatment with C5a. Cell count did not change following 30 min 0.001, 0.01, 0.1, 1, or 10 nM treatment in C5a. One-way ANOVA with Dunnett’s post hoc test were used for statistical analysis. ns = non significant.

The level of caspase activation, PI staining, and overall cell staining was determined following treatment of cells with 0 (control), 0.001, 0.01, 0.1, 1, and 10 nM C5a for 30 min. The level of caspase activation was increased following 30 min treatment of cells with 10 nM C5a and H_2_O_2_ (Figure 1a). The level of cell death as indicated by PI staining was significantly elevated only in the H_2_O_2_ positive control (Figure 1b). The cell count did not differ between any of the treatment conditions (Figure A1). This demonstrated an increase in caspase 3/7 activity, but not apoptosis, following a 30 min treatment of PC12 cells with 10 nM C5a.

**Figure 2:**
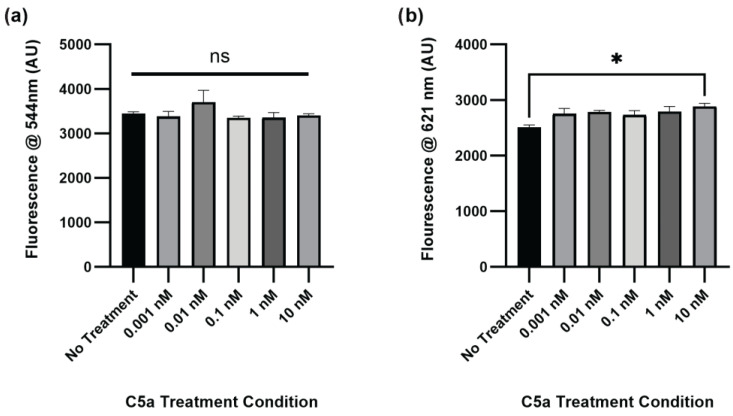
Caspase Activity and PI Staining following 24 h Treatment with C5a. (**a**) The level of caspase activation was measured by detecting the fluorescence at an excitation wavelength of 495 ± 20 nm and an emission wavelength of 544 ± 25 nm. There was no significant change in caspase activation at this timepoint. (**b**) The level of PI staining was determined by measuring the fluorescence using an excitation wavelength of 586 ± 15 nm and a read wavelength of 621 ± 25 nm. There was increased cell death in the 10 nM C5a treatment relative to the no-treatment condition. One-way ANOVA with Dunnett’s post hoc test were used for statistical analysis. * *p* < 0.05. ns = non significant. *n* = 3.

**Figure A2:**
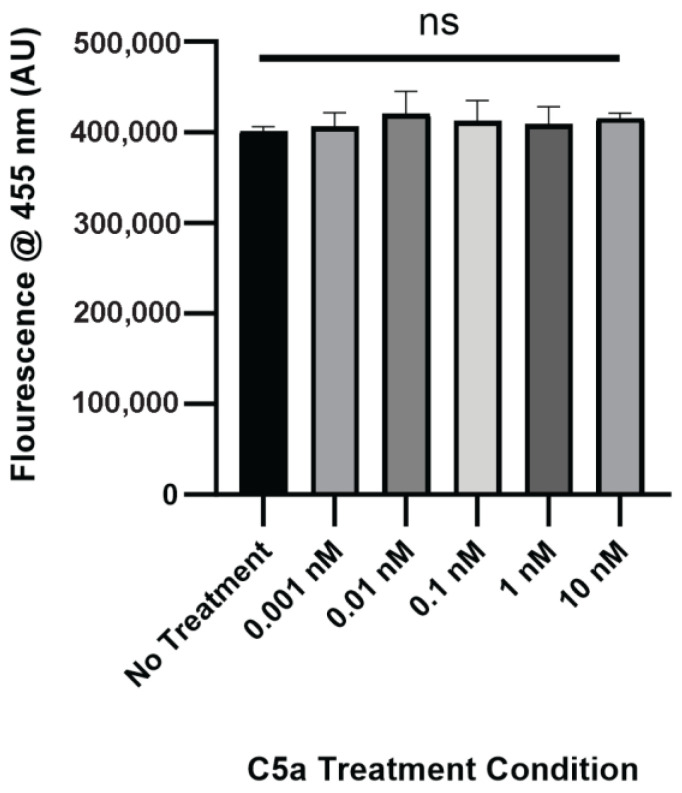
Nuclear Staining following 24 h Treatment with C5a. Cell count did not change following 24 h 0.001, 0.01, 0.1, 1, or 10 nM treatment in C5a. One-way ANOVA with Dunnett’s post hoc test were used for statistical analysis. ns = non significant.

The level of caspase activation, PI staining, and overall cell staining was also determined following treatment of PC12 cells with C5a for 24 h. There was no significant change in caspase activation for any of the treatment conditions relative to the no-treatment control (Figure 2a). The level of cell death was significantly elevated in the 10 nM treatment (Figure 2b). The cell count did not differ between any of the treatments (Figure A2). The H_2_O_2_ positive control was not used at the 24 h timepoint because following the H_2_O_2_ treatment the cells were completely degraded and there was no signal detectable above background. This demonstrated an increase in cell death, but not caspase 3/7 activity, following a 24 h treatment of PC12 cells with 10 nM C5a.

### 2.2. PMX-53 Inhibits C5a-Induced Caspase Upregulation

**Figure 3:**
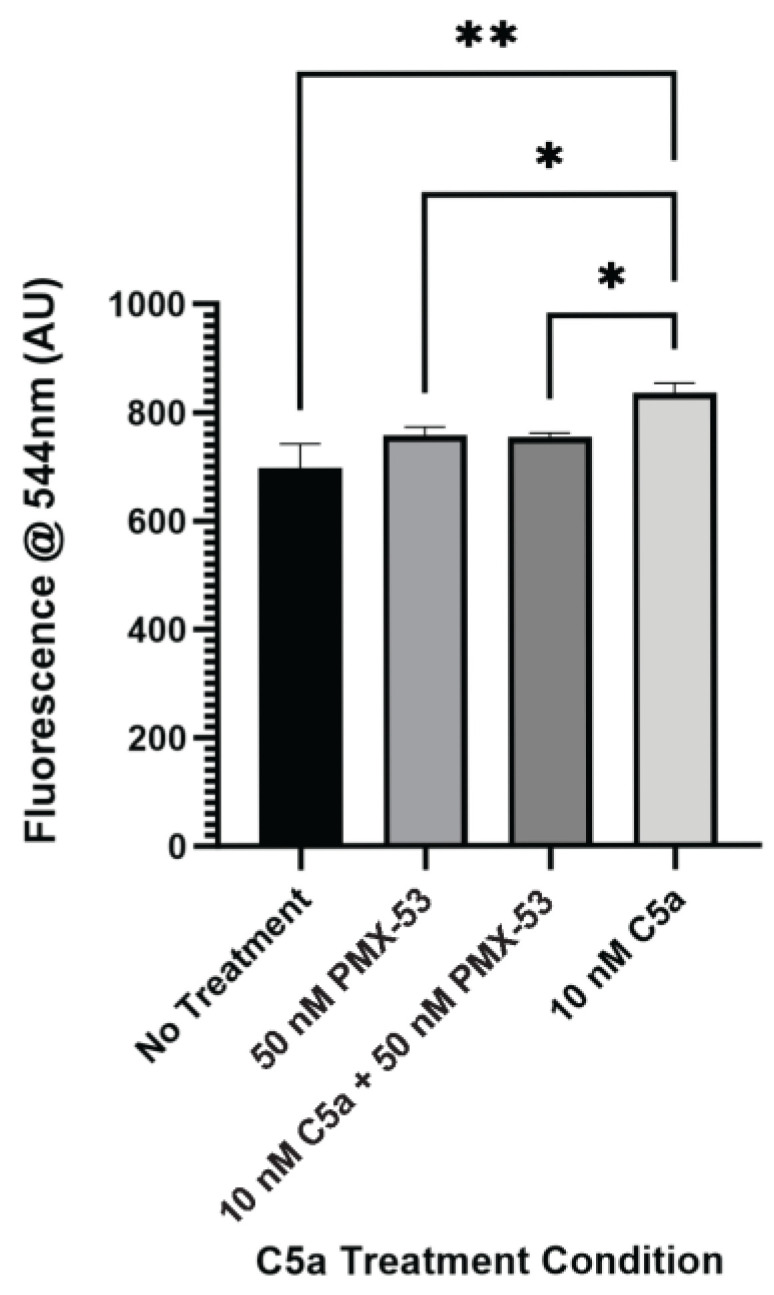
Effect of C5aR Inhibitor on Caspase Activation. The level of caspase activation following a 30 min incubation with various concentrations of C5a and 50 nM PMX-53 was examined. This was measured by detecting the fluorescence at an excitation wavelength of 495 ± 20 nm and an emission wavelength of 544 ± 25 nm. There was an increase in the level of caspase activation for the 10 nM C5a treatment compared to the no treatment, 50 nM PMX-53, and 10 nM C5a + 50 nM PMX-53 treatments.. Treatment with 50 nM PMX-53 alone or 10 nM C5a/50nM PMX-53 did not induce an upregulation in caspase activation relative to the control. One-way ANOVA with Dunnett’s post hoc test were used for statistical analysis. * *p* < 0.05, ** *p* < 0.01. *n* = 3.

**Figure A3:**
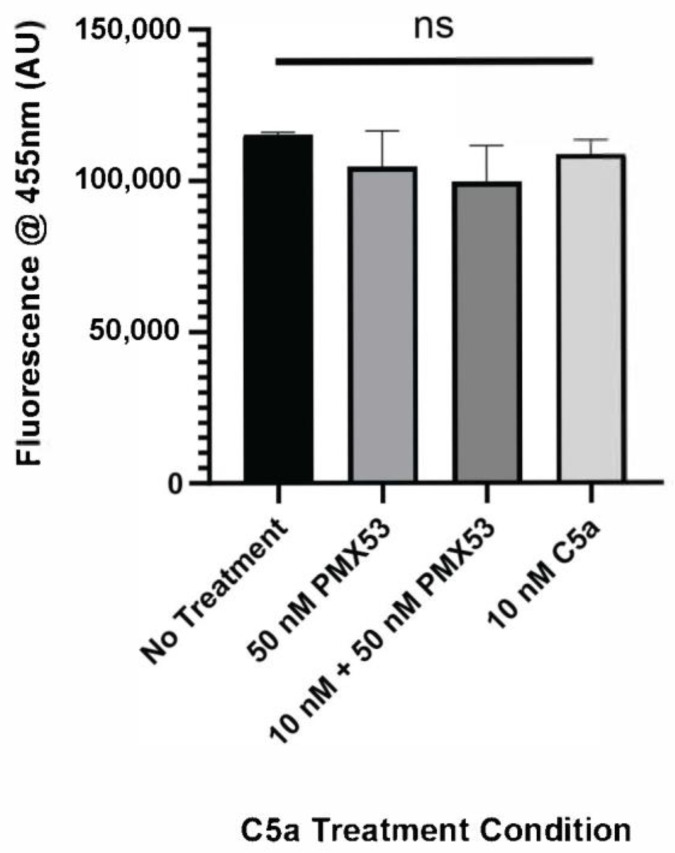
Effect of C5aR Inhibitor on Caspase Activation. Pre-treatment of PC12 cells with 50 nM PMX-53 and treatment with C5a for 30 min did not impact cell count. One-way ANOVA with Dunnett’s post hoc test were used for statistical analysis. ns = non significant.

After identifying increased caspase activation and PI staining as a result of 10 nM C5a treatment, the C5aR inhibitor, PMX-53, was utilized to identify whether this receptor is involved in the cell death response mediated by C5a. PC12 cells were treated with 10 nM C5a or 50 nM PMX-53 alone or in combination (50 nM PMX-53 + 10 nM C5a). Following incubation in 10 nM C5a, caspase activation was increased compared to the no-treatment condition. This response was inhibited by pre-treatment with 50 nM PMX-53, a C5aR inhibitor (Figure 3). Treatment with 50 nM PMX-53 alone did not induce an increase in caspase activation. The cell count did not differ between the different treatment conditions (Figure A3). This demonstrated that the increase in caspase 3/7 activity following 30 min treatment of PC12 cells with 10 nM C5a was C5aR-dependent.

### 2.3. C5a Induces Inflammatory Signaling in PC12 Cells

**Figure 4:**
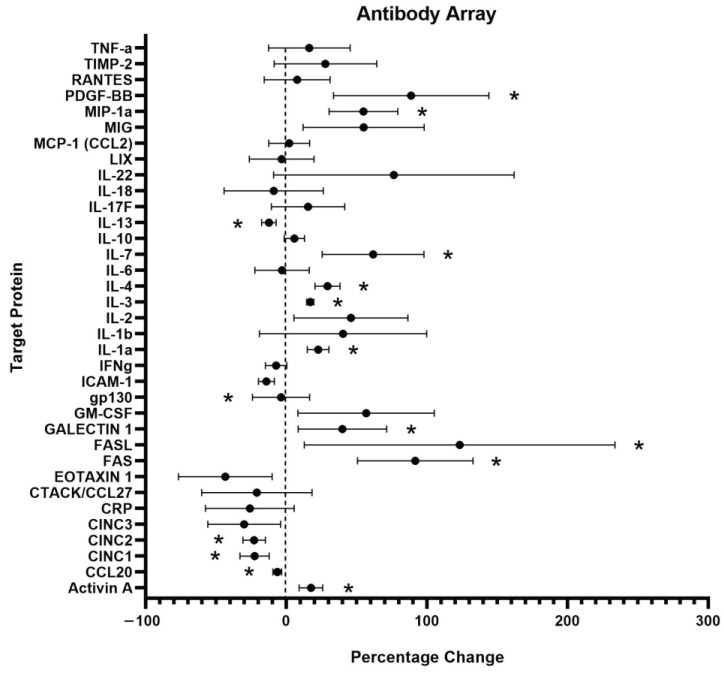
Inflammatory Antibody Array Analysis. An antibody array labelled with 36 unique protein targets was used to examine the levels of inflammatory protein produced by PC12 following a 24 h incubation in 10 nM C5a. n = 3. An unpaired *t*-test used for statistical analysis. For significance information, see Table 1 and Table 2. * *p* < 0.05. *n* = 3.

The level of inflammation induced by PC12 cell exposure to C5a was measured by detecting the levels of various inflammatory cytokines related to inflammatory signaling using an antibody array (Figure 4). The cells were examined following a 24 h incubation in 10 nM C5a and compared to a no-treatment control. There was a total of 10 proteins that were determined to be upregulated and 5 that were significantly downregulated following treatment with 10 nM C5a (Table 1 and Table 2). The general function of the individual proteins is detailed in Table 1 and Table 2. The general functions of the proteins that were upregulated are apoptosis, inflammatory processes, and cell health/proliferation. The proteins that were downregulated were generally involved in chemotaxis, immune cell differentiation, and anti-inflammation.

### 2.4. Complement C5a Induces Protein Kinase Signaling in PC12 Cells

**Figure 5:**
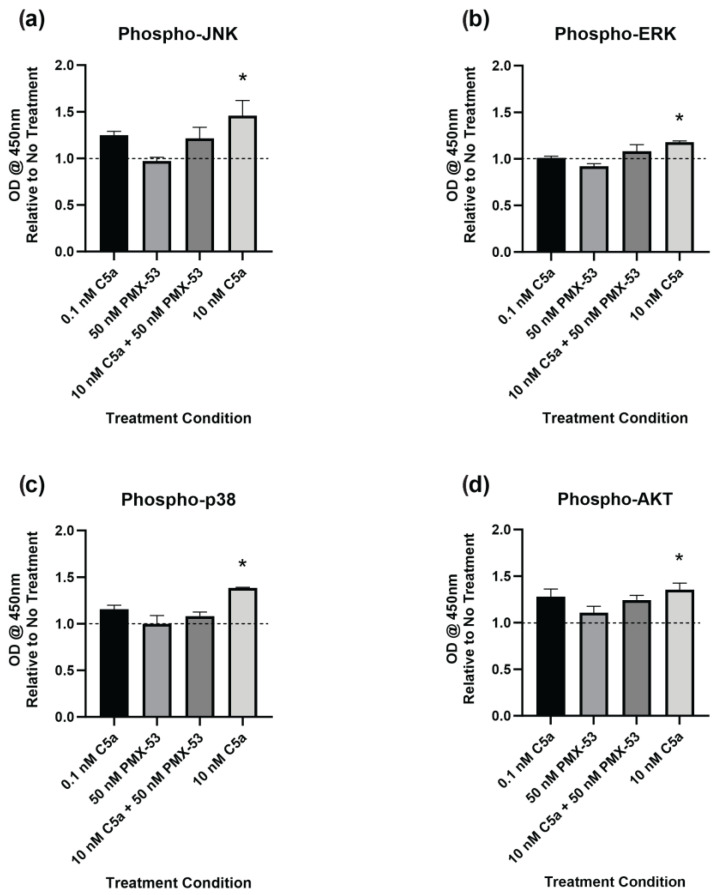
Phosphorylated Protein Kinase Pathway Analysis. Phosphorylation of (**a**) JNK1/2, (**b**) ERK1/2, (**c**) p38 (pT180/pY182), and (**d**) AKT (PI3K) was analyzed by ELISA. The cells were incubated in 0.1 nM C5a, 10 nM C5a, 10 nM C5a + 50 nM PMX-53, and 50 nM PMX-53. The OD at 450 nm was determined, and the data were graphed relative to the no-treatment control (dashed line). Following treatment with 10 nM C5a, the activations of JNK1/2, ERK1/2, p38, and AKT were all upregulated relative to the control. * *p* < 0.05. *n* = 3.

The level of phosphorylation of target proteins in specific intracellular signaling pathways was determined using ELISA kits. This was performed to better understand the signaling pathways inducing caspase activation and the apoptotic response. The cells were examined following 30 min of incubation with C5a (0.1 nM, 10 nM), PMX-53 inhibitor (50 nM) alone, or in combination (10 nM C5a + 50 nM PMX53). Increased phosphorylation of JNK1/2 (Figure 5a), ERK1/2 (Figure 5b), p38 (Figure 5c), and AKT (Figure 5d) was observed following treatment with 10 nM C5a for 30 min. Pre-treatment of PC12 cells with 50 nM PMX-53 inhibited this response for all analytes. Neither the 0.1 nM C5a nor the 50 nM PMX-53 treatment alone showed an increased level of phosphorylation. These results demonstrate a C5aR-dependent increase in JNK1/2, ERK1/2, p38, and AKT phosphorylation following 30 min treatment of PC12 cells with 10 nM C5a.

## 3. Discussion

### 3.1. C5a and Apoptosis

Inflammation in sepsis is known to induce adrenal gland damage; furthermore, complement C5a has been suggested to induce apoptosis in PC12 cells [23,61,62]. The current study further investigated and characterized the mechanisms involved in C5a-induced apoptosis.

The viability of PC12 cells was decreased following incubation with elevated concentrations of C5a (10 nM). Incubations with lower (≤1 nM) C5a concentrations did not induce a decrease in cell viability. These results are interesting since concentrations of C5a are measured at >10 nM in septic conditions [63]. The decrease in cell viability at 10 nM of C5a provides an argument for the cytotoxic effect of C5a on adrenal tissue in sepsis [22]. The caspase activation assay showed a significant increase in the activation of caspases following 10 nM C5a incubation at the 30 min timepoint. This increase in caspase activation is in accordance with what has been previously reported in the literature. Previous studies show that C5a induces caspase 3/7 activation in PC12 cells and that this apoptotic response was inhibited with a caspase inhibitor [23]. The current study demonstrates a novel result where this caspase activation was also inhibited by pre-treatment with the C5aR inhibitor PMX-53. This finding indicates that the apoptotic response is dependent on C5aR signaling. PI staining has previously been used to examine cell viability in PC12 cells; however, only treatment with 10 nM C5a has been examined [23]. In addition, the previous studies that implemented PI to study C5a-induced apoptosis in PC12 cells only presented images of the staining without any quantified data analysis. Despite this lack of quantitation, the increase in propidium iodide staining seen in the paper following exposure to 10 nM for 30 min is drastic, further suggesting an apoptotic response of PC12 cells by 10 nM C5a [23]. Also of note, only the 30 min timepoint was previously examined, and the long-term effects of this incubation were not examined. Overall, the results of this study are in accordance with, and expand upon, the current literature on C5a cytotoxicity in adrenal cells.

Incubation with 10 nM C5a for 24 h induced an increase in FAS, FASL, and activin A expression in PC12. The increased expressions of FAS and FASL were the largest significant changes in the assessed proteins. The FAS receptor and its ligand (FASL) have been repeatedly shown to be involved in apoptosis in PC12 cells [64,65,66]. The increase in FAS/FASL in PC12 cells is supported by observations reported in other cell lines. C5a plays a role in activating the FAS/FASL axis, with C5a-induced FAS playing a role in brain vascular cell apoptosis and caspase activation in an experimental lupus model [25]. Additionally, C5a exposure was shown to increase the expression of the FAS ligand in B-lymphoblasts [67].

Activin A has also been repeatedly shown to induce an apoptotic response in numerous cell lines [68,69,70]. In NGF-differentiated PC12 cells, however, it had a neuroprotective and anti-apoptotic effect [71,72]. Activin A has been shown to activate p38 signaling in undifferentiated PC12 cells, which, as discussed later, has potential implications for apoptotic signaling [73]. To date, activin A and apoptosis have not been extensively examined in undifferentiated PC12 cells and are a potential area for further investigation.

While there are some other studies examining C5a cytotoxicity, they primarily examine the effect on immune cells; however, there is an increasing body of research demonstrating the cytotoxic effects of septic levels of C5a in non-immune tissues. To date, these effects at elevated C5a concentrations have been examined mainly in endothelial tissue models [25,74]. Overall, the results of this study help to corroborate the growing body of evidence suggesting an apoptotic effect of elevated concentrations of C5a, with the induction of apoptotic signaling mediated by C5aR.

### 3.2. C5a and Cellular Signaling

The activation of JNK1/2, ERK1/2, p38, and AKT1/2/3 pathways was increased following treatment with 10 nM C5a for 30 min. The phosphorylation ELISA method was used because the activity of these pathways is mediated by post-transcriptional modifications, namely phosphorylation, on established sites of the proteins [75,76]. Characterizing the level of phosphorylated proteins allowed for the determination of the levels of activation of each of the respective pathways.

The activation of all four pathways was inhibited by pre-treatment with 50 nM PMX-53, a C5aR inhibitor, demonstrating that C5a signaling occurred in a C5aR-dependent manner. Our data are consistent with previous reports that the activation of all four of the studied protein kinase pathways by C5a is dependent on the C5a/C5aR pathway, while C5a/C5L2 does not appear to result in activation of these pathways [77,78,79,80,81]. As previously discussed, the function of C5L2, which is not a GPCR, is not clearly elucidated, and more research is needed to understand its role in C5a signaling.

#### 3.2.1. JNK

A member of the MAPK family, the JNK pathway is an important regulator of cell function, primarily apoptosis, gene transcription, and cellular survival [82]. It is also notable for its role in modulating neuronal function [83]. In PC12 cells, JNK is associated with the induction of apoptosis following exposure to various stressors [84,85,86]. In some models of apoptosis in PC12 cells, the JNK and p38 pathways are both necessary for the induction of apoptosis [87]. In septic conditions, JNK has been identified as a target for drug treatments due to the hyperactivation of JNK and its role in stimulating inflammation and apoptosis [88,89]. The apparent pro-apoptotic effects of JNK activation may be a potential explanation for the apoptosis observed in PC12 cells in this study.

#### 3.2.2. ERK

The ERK kinases are a member of the MAPK family and are generally involved in cell growth, proliferation, and differentiation [90]. Additionally, ERK signaling is known to induce signaling in response to inflammatory stimuli and has been implicated in the Fas-induced cell death pathway in certain cell types [91,92,93,94]. In contrast, ERK signaling may be activated to counteract cell death pathways and promote cell proliferation and survival [95]. In accordance with this previous literature on immune cells, ERK activation was increased in response to C5a [96].

#### 3.2.3. p38

The third member of the MAPK family, p38, plays an important role in various cellular processes, including inflammation, proliferation, and apoptosis [97]. In PC12 cells, p38 has been shown to be involved in the apoptotic response to various stressors [98,99,100]. In addition, p38 has been shown to be involved in the recruitment and cleavage of caspases in cell death [101,102]. Furthermore, p38 is involved in the previously discussed mitochondrial-dependent pathway of apoptosis [103]. The inflammatory response in sepsis is largely dependent on p38 activation, which in turn has been shown to be activated by C5a [104,105,106]. p38, along with the other MAPK pathways, plays an important role in inflammation regulation through the production of various cytokines and other inflammatory molecules [107]. This role in inflammatory molecule production may help to explain the results seen in the antibody array (Table 1).

#### 3.2.4. AKT

AKT (also known as PKB) is a serine/threonine kinase involved in cell cycle regulation, metabolism, apoptosis, and the regulation of various gene targets [108]. In PC12 cells, AKT is often upregulated in response to stressors [109,110,111]. In other tissues, C5a-induced MAPK and AKT activation is linked to damage to cardiac and liver tissues in various stress states and has been argued to provide a protective effect [112,113]. Moreover, in septic conditions, C5a-induced activation of MAPK and AKT signaling has been linked to cardiac dysfunction [114]. When this activation was inhibited by a p38 inhibitor, inflammatory cytokine release was decreased, suggesting a role for MAPK and AKT in inflammatory signaling in non-immune tissues. AKT has also been shown to induce Cyclin E2, and this may be a mechanism by which Cyclin E2 was upregulated following C5a treatment in this study [115].

#### 3.2.5. Intercellular and Inflammatory Signaling

Treatment of PC12 cells with 10 nM C5a induced an increase in the production of 10 inflammatory proteins (Table 1) and a decrease in the production of 5 inflammatory proteins (Table 2). FAS and FASL have previously been shown to be upregulated by C5a in other cell types and are involved in the FAS-apoptotic pathway. Various immune cell types have been shown to upregulate the levels of the identified inflammatory cytokines in response to C5a, including IL-1α, IL-4, and MIP-1α. Other cytokines showed differential responses in PC12 cells than in immune cells, such as IL-13, which was downregulated but has been shown to be upregulated in response to C5a/Il-3 treatment in basophils [116].

#### 3.2.6. Chemotaxis

The decrease in expression of chemotaxis-associated proteins is systematically observed in this experimental model. It is worth noting that C5a is a potent chemotactic agent for immune cells and is commonly cited as one of the primary effector functions of C5a in neutrophils and macrophages [117,118,119]. C5a has also been shown to aid in CCL20-dependent T-cell recruitment [120].

Interestingly, some of the proteins that are downregulated, such as IL-13 and CCL20, are upregulated in other cell types following exposure to C5a [117,121]. This contradiction helps to support the argument for differential C5a signaling effects across cell types. This has been described in the effect of C5a on mast cells, where the induced signaling effects were shown to vary between different variants of mast cells [122]. Recently, this line of research was expanded to suggest that C5a responsiveness is cell-type-dependent [123]. This differential response may help to explain differences in the expression levels of various proteins in cell lines following exposure to C5a.

### 3.3. Clinical Relevance and Future Directions

Currently, there are a small number of drugs targeting complement C5a and C5aR. Avacopan is a C5aR antagonist that was approved in 2021 in the United States for the treatment of antineutrophil cytoplasmic antibody (ANCA)-associated vasculitis [124]. Eculizumab is an antibody that binds to complement C5, inhibiting the cleavage of C5 to C5a and C5b, and is approved for the treatment of paroxysmal nocturnal hemoglobinuria [125]. Therapeutics targeting C5a in sepsis have been explored in animal models but have not been examined extensively in human studies. However, Eculizumab was used in a study investigating ICU patients with severe COVID-19 and increased the survival rate from 62.2% (48.1–76.4%) to 82.9% (95% CI: 70.4–95.3%). The results of the current study, coupled with the increased usage of therapeutics targeting C5a and C5aR, suggest a promising potential area for investigation in the treatment of sepsis. Future studies should examine a potentially protective effect of inhibiting C5a/C5aR signaling in septic conditions.

## 4. Materials and Methods

### 4.1. Materials

Dulbecco’s modified Eagle’s medium (DMEM), bovine calf serum, and equine serum were purchased from Medi-Res Corporation (Sudbury, ON, Canada). Rat recombinant C5a (Asp1-Arg77) was purchased from Abbexa (Cambridge, UK). The C5a inhibitor PMX-53 was purchased from Cayman Chemical Company (Ann Arbor, MI, USA). Propidium Iodide (PI), the CellEvent™ Caspase-3/7 Detection Reagent, NucBlue™ nuclear probe, FluoroBrite™ DMEM, and the white-walled 96-well tissue-culture treated plate were purchased from Thermo Fisher Scientific (Nepean, ON, Canada). The rat inflammation array (C3) was purchased from Raybiotech (Peachtree Corners, GA, USA). The phosphorylation arrays were purchased from Thermo Fisher Scientific. All other reagents were purchased from Medi-Res Corporation.

### 4.2. Cell Culture

PC12 cells (Dr. Sushil Mahata, Department of Medicine, University of California, San Diego, CA, USA) were cultured in DMEM supplemented with 5% equine serum, 5% bovine calf serum, and gentamycin sulfate (50 µg/mL). The cells were maintained in a humidified incubator at 37 °C in an atmosphere of 5% CO_2_. Prior to experimentation, cells were transferred to FluoroBrite™ DMEM containing charcoal-treated serum.

### 4.3. C5a Treatment

The C5a was reconstituted to a concentration of 2.5 μM in accordance with the manufacturer’s specifications. In the experiments requiring treatments, PC12 cells were treated with varied concentrations of C5a (0.001, 0.01, 0.1, 1, and 10 nM) and a no-treatment control. The cells were treated for either 30 min or 24 h, depending on the experimental protocol.

### 4.4. Caspase Activation and Cell Death Assay

Cells were seeded at a density of 2 × 10^4^ cells/well in white-walled 96-well plates. The cells were treated with C5a for specific treatment times: (1) 30 min, to examine the level of caspase activation following treatment; and (2) 24 h, which examined potential continued caspase activation and late-stage apoptosis in PC12 cells. A subset of samples was also pre-treated with 10 nM PMX-53 for 30 min. The cells were incubated with the Caspase 3/7 detection reagent, PI, and nuclear probe in accordance with the manufacturer’s protocol. A positive H_2_O_2_ control was used to induce cell death, and the data were compared to the no-treatment negative control. The well fluorescence was measured using a Biotek Cytation 5 Multimode Reader (Agilent Technologies, Santa Clara, CA, USA).

### 4.5. Antibody Array

Cells were seeded at a density of 5 × 10^5^ cells/well in 6-well plates. The cells were treated with 10 nM C5a for 24 h. The media were aspirated from the plate, and the cells were trypsinized, resuspended in media, and then transferred to a 1.5 mL tube. The protein cell lysate was prepared by centrifuging the sample at 1000× *g* for 5 min at 4 °C and aspirating the supernatant. The pellet was resuspended in 100 uL of RIPA lysis buffer (150 mM NaCl, 1% Nonidet P-40, 0.5% DOC, 0.25% SDS, 50 mM Tris [pH = 7.4]) containing a protease inhibitor (87785, Thermo Fisher Scientific) and stored at −80 °C overnight. The cell lysate was thawed and then sonicated (Branson 150, Thermo Fisher Scientific) for 4 s (5×), with a 1 min rest between each pulse. The samples were then incubated for 15 min on ice, with vortexing every 3 min. The samples were centrifuged for 10 min at 20,000× *g* at 4 °C. The protein lysate was then analyzed using the antibody array in accordance with the manufacturer’s specifications. The array was imaged using the ChemiDoc Imaging System (Biorad, Hercules, CA, USA) and analyzed using ImageJ software (Version 1.54g) with the DotBlot Plugin (http://image.bio.methods.free.fr/dotblot.html [accessed on 12, April 2024]).

### 4.6. Phosphorylation ELISA

PC12 cells were treated with 0.1 and 10 nM C5a, and a subset of cells was also pre-treated with 50 nM PMX-53 for 30 min. The levels of phosphorylated AKT, JNK1/2, ERK1/2, and p38 were determined following the manufacturer’s protocol. The optical density at 450 nm with a reference wavelength of 650 nm was determined using the Biotek Cytation 5 Multimode Reader.

### 4.7. Statistical Analysis

Statistics were performed using GraphPad Prism software (Version 10.0.3) (La Jolla, CA, USA) and were analyzed using one-way analysis of variance analysis (ANOVA) with Dunnett’s post hoc test or unpaired *t*-test. All data are presented as the mean ± standard error of mean (SEM) of at least three independent experiments.

## 5. Conclusions

The current study investigated the impact of C5a on PC12 signaling and apoptosis. Incubation with 10 nM C5a triggered caspase activation in PC12 cells through a C5aR-dependent mechanism. Exposure to septic levels of C5a (10 nM) induced C5aR-dependent JNK/MAPK, ERK/MAPK, p38/MAPK, and AKT (PI3K) signaling. Furthermore, exposure to 10 nM C5a for 24 h induced an increase in the expression of proteins associated with cell survival, inflammatory processes, and apoptosis and a decrease in the expression of proteins associated with chemotaxis, adhesion, and anti-inflammatory effects. These results provide insight into the mechanism of C5a-induced apoptosis and cellular signaling in PC12 cells. These findings suggest that elevated levels of complement C5a may play an important role in the mechanism of adrenal dysfunction in sepsis.

## Figures and Tables

**Table 1:** Upregulated Inflammatory Proteins After C5a Treatment.

Protein	Percentage Change	SEM (%)	Significant (*p* < 0.05)	Function
Activin A	17.59	4.868	Yes	Cell Cycle Progression, Cell Proliferation, and Apoptosis [47]
FAS	91.74	23.7	Yes	Induction of Apoptosis [48]
FASL	99.56	50.96	Yes	
GALECTIN 1	39.95	11.54	Yes	Regulation of Cell Cycle [49]
IL-1*α*	22.68	4.439	Yes	Alarmin and Inflammatory Process [50]
IL-3	17.01	1.369	Yes	Inflammatory processes, Cell Growth, and Differentiation [51]
IL-4	29.42	5.13	Yes	Proliferation and Cell Survival [52]
IL-7	61.77	19.08	Yes	Proliferation and Cell Survival [53]
MIP-1a	54.91	12.89	Yes	Immune Cell Activation and Chemotaxis [54]
PDGF-BB	88.81	20.92	Yes	Proliferation, Immune Cell Activation, and Differentiation [55]

**Table 2:** Downregulated Inflammatory Protein After C5a Treatment.

Protein	Percentage Change	SEM (%)	Significant (*p* < 0.05)	Function
CCL20	−6.356	1.787	Yes	Immune Migration and Chemotaxis [56]
CINC1	−22.5	6.018	Yes	Neutrophil Chemoattractant [57]
CINC2	−22.68	4.599	Yes	Immune Cell Recruitment and Chemotaxis [58]
ICAM-1	−14.18	3.261	Yes	Cell Adhesion and Immune Cell Recruitment [59]
IL-13	−12.33	2.959	Yes	Anti-inflammatory Effects [60]

## Data Availability

The raw data supporting the conclusions of this article will be made available by the authors on request.
